# The impact of breast surgery and systemic therapy on the survival of patients with de novo stage IV breast cancer

**DOI:** 10.1007/s12282-025-01675-w

**Published:** 2025-02-02

**Authors:** Eriko Tokunaga, Yumiko Koi, Wakako Tajiri, Chinami Koga, Hideki Ijichi, Sayuri Akiyoshi, Junji Kawasaki, Yoshiaki Nakamura, Kenichi Taguchi, Masahiro Okamoto

**Affiliations:** 1 Department of Breast Oncology, NHO Kyushu Cancer Center, 3-1-1 Notame, Minami-Ku, Fukuoka, 811-1395 Japan; 2 Departments of Pathology, NHO Kyushu Cancer Center, 3-1-1 Notame, Minami-Ku, Fukuoka, 811-1395 Japan

**Keywords:** Breast cancer, De novo stage IV, Surgery, Systemic therapy, Tumor subtype

## Abstract

**Background:**

Systemic therapy (ST) is essential for de novo stage IV breast cancer (BC). Stage IV BCs are highly heterogeneous, and it seems inappropriate to treat all de novo stage IV BCs equally. The survival benefit of surgery for primary sites in patients with de novo stage IV BC remains inconclusive.

**Patients and methods:**

We investigated 220 patients with clinical de novo stage IV BC. The relationship between primary site surgery and overall survival (OS) was analyzed. Factors such as tumor subtype, timing of surgery, and efficacy of ST were also evaluated.

**Results:**

The median follow-up time was 37.9 (0.5–201.7) months. In the total cohort, the median OS of the patients with and without primary site surgery was 70.5 months (95% confidence interval [CI] 58.4–107.3) and 42.7 months (95% CI 35.7–48.8), respectively. The OS was significantly longer in patients who underwent primary site surgery, especially in the hormone receptor (HR) + /HER2- and HER2 + subtypes, but not in the triple-negative subtype. OS prolongation was significant in patients who underwent surgery ≥ 24 months after the first diagnosis and in whom the first-line ST was effective for ≥ 24 months. Primary site surgery was a good prognostic factor in both univariate and multivariate analyses.

**Conclusions:**

The OS was significantly longer in patients with de novo stage IV BC who underwent primary site surgery than in those who did not undergo surgery. Our results suggest that the tumor subtypes, efficacy of ST, and timing of surgery influenced the benefits of surgery.

**Supplementary Information:**

The online version contains supplementary material available at 10.1007/s12282-025-01675-w.

## Introduction

Metastatic breast cancer (MBC) is considered incurable, and patients with MBC are basically treated with systemic therapy (ST) to prolong the survival time, relieve symptoms, and improve the quality of life (QOL). The precise ST regimen is selected based on the tumor subtypes, defined by hormone receptor (HR) and HER2 status, as well as by other biomarkers, such as PD-L1 and germline *BRCA1/2* gene mutations [1, 2].

The clinical course of MBC is variable because of the large variations in growth rate, aggressiveness, and responsiveness to ST. The survival of patients with MBC is strongly related to responsiveness to ST. For patients with de novo stage IV breast cancer (BC), ST is also the most important treatment strategy. Removal of the primary tumor in patients with de novo stage IV BC improves the QOL in selected patients with controlled systemic disease with ST. However, the survival benefit of surgery for the primary site is inconclusive [1, 2].

Some retrospective studies have shown an association between surgery for primary tumors and an improved survival in patients with de novo stage IV BC [3–10]. These studies were highly heterogeneous in terms of patient background, systemic therapy, timing of surgery, and other clinical features, so the results might have been influenced by significant biases [11, 12].

To clarify the clinical significance of surgery for the primary sites of de novo stage IV BC, several prospective randomized trials have been performed. Only one study showed a positive result [13]; however, others failed to confirm the survival benefit of resection of the primary tumor [14–16]. In a randomized trial conducted in Japan, the survival benefit of primary tumor resection was not revealed [17].

Recently, an increasing number of new drugs have been approved for the treatment of BC. For MBC, prolongation of the duration of response to ST could prolong the overall survival (OS). The impact of surgery for primary sites in de novo stage IV BC patients could be influenced by the efficacy of ST. However, in previous studies, there are few discussions regarding the association between the efficacy of ST and the survival benefit of primary tumor resection.

Therefore, we focused on the comprehensive evaluation of the clinical significance of surgery for the primary site, along with the tumor subtype, the efficacy of ST, and the surgical timing in patients with de novo stage IV BC.

## Patients and methods

### Patient population

A total of 220 patients with clinical de novo stage IV BC who were diagnosed between 2009 and 2019 at the Department of Breast Oncology, National Hospital Organization (NHO) Kyushu Cancer Center, were included in this study. Clinical data were obtained from the patients’ medical records. The anatomical AJCC/UICC TNM classification and stage grouping were used.

This study was approved by the institutional review board of our hospital and conducted in accordance with the Declaration of Helsinki. We obtained written informed consent from each patient before the start of the treatment and sample acquisition.

### Pathological examinations

All pathological examinations were performed by experienced pathologists at our hospital. The expression of the estrogen receptor (ER) and progesterone receptor (PgR) was regarded as positive if the nuclear expression was ≥ 1%. Hormone receptor (HR +) positivity was defined as ER + and/or PR + . The HER2 status was evaluated according to ASCO/CAP recommendations [18]. If ER and/or PgR were positive, the case was defined as (HR +). The tumor subtypes were divided into four groups (HR + /HER2-, HR + /HER2 + , HR−/HER2 + , and triple-negative [TN; ER−, PgR−, and HER2−]) or three groups (HR + /HER2−, HER2 + [HR + /HR−], and TN).

### Statistical analyses

Statistical analyses were performed using the JMP software package (version 14.0; SAS Institute Inc., Cary, NC, USA). Associations between clinicopathological characteristics were assessed using the χ^2^ test. The OS was defined as the time from the date of the diagnosis of stage IV breast cancer to death. Survival curves were plotted using the Kaplan–Meier method, and the association between the survival and each variable was determined using the log-rank test. The Cox proportional hazards model was used for the multivariate analysis of survival data. Differences were considered statistically significant at P < 0.05.

## Results

### Patients' characteristics

A total of 220 patients were included in this study. Patient characteristics are shown in Table 1. The median age of the patients was 57 (range: 31–86) years old. Seventy-one patients (32.3%) were pre/perimenopausal, and 143 (65.0%) were postmenopausal. In terms of tumor subtypes, 113 (51.4%) were HR + /HER2−, 33 (15.0%) HR + /HER2 + , 42 (19.1%) HR−/HER2 + and 24 (10.9%) HR−/HER2− (TN). Visceral metastases were detected in 120 (54.5%) patients at the first diagnosis. Forty-nine patients (22.3%) had liver metastases, and 69 patients (31.4%) had only bone metastases. The number of metastatic sites was 1 in 140 patients (63.6%), 2 in 54 (24.5%), and ≥ 3 in 26 (11.8%). As the first treatment, 101 (45.5%) patients had received endocrine therapy; 59 (26.8%), 45 (20.5%), and 7 (3.2%) patients received chemotherapy alone, chemotherapy + anti-HER2 therapy, and anti-HER2 therapy alone, respectively. Eight (3.6%) patients underwent surgery at the primary site as the first treatment. Throughout the treatment course, surgery for the primary tumor was performed in 61 patients (27.7%). Surgery was performed immediately after the diagnosis in 8 patients (13.1%) and within 6 months of starting treatment in 10 patients (16.4%), 6≦ < 12 months in 15 patients (24.6%), 12≦ < 24 months in 11 patients (18.0%), and after ≥ 24 months in 17 patients (27.9%).

**Table 1:** Clinicopathological characteristics of the patients

Factors	n (%)
Age (years)	
Median (range)	57 (31–86)
Menopausal status	
Pre/peri	71 (32.3)
Post	143 (65.0)
Unknown	6 (2.7)
Tumor subtype	
HR + /HER2-	113 (51.4)
HR + /HER2 +	33 (15.0)
HR-/HER2 +	42 (19.1)
TN	24 (10.9)
Unknown	8 (3.6)
Viscetal metastasis at the first diagnosis of breast cancer	
No	100 (45.5)
Yes	120 (54.5)
Metastatic site	
Liver	49 (22.3)
Visceral other than liver	71 (32.3)
Bone only	69 (31.4)
Others	31 (14.1)
Distant lymph nodes only	20 (9.1)
Bone and distant lymph nodes	7 (3.2)
Bone and skin	2 (0.9)
Skin and distant lymph nodes	1 (0.5)
Skin only	1 (0.5)
Number of metastatic sites	
1	140 (63.6)
2	54 (24.5)
≥ 3	26 (11.8)
First treatment	
Endocrine therapy	101 (45.9)
Chemotherapy	59 (26.8)
Chemotherapy + anti-HER2 therapy	45 (20.5)
Anti-HER2 therapy	7 (3.2)
Surgery	8 (3.6)
Surgery for the primary lesion	
No	159 (72.3)
Yes	61 (27.7)
Timing of the surgery	
Just after the diagnosis	8 (13.1)
Within 6 months	10 (16.4)
6–12 months	15 (24.6)
12–24 months	11 (18.0)
More than 24 months	17 (27.9)

HR: hormone receptor, TN: triple-negative distant lymph node metastases; cervical lymph nodes, mediastinal lymph node, contralateral axillary lymph node, pelvic lymph nodes, etc.

### Relationships between the clinicopathological characteristics and surgery for primary site

Table 2 shows the relationships between clinicopathological characteristics and surgery at the primary site. There were no relationships among age, menopausal status, and performance of surgery (surgery/no surgery). Surgery was performed differently, depending on the year of the first diagnosis. Patients without visceral metastases were more likely to undergo surgery at the primary site than those with visceral metastases (P = 0.0405). Patients with 1 metastatic site underwent surgery more often than those with ≥ 2 metastatic sites (P = 0.0037). Patients with HR−/HER2 + and TN underwent surgery more frequently than those with other subtypes.

**Table 2:** Relationships between the clinicopathological characteristics and surgery for the primary site

Factors	No surgery (n = 159)	Surgery (n = 61)	P-value
Age (years)			
Mean ± SE	57.3 ± 1.00	54.2 ± 1.61	0.1093
Menopausal status			
Pre/perimenopause	46 (28.9)	25 (41.0)	0.2154
Postmenopause	108 (67.9)	35 (57.4)	
Unknown	5 (3.1)	1 (1.6)	
Year of the diagnosis			
2000–2004	31 (19.5)	9 (14.8)	0.0472
2005–2009	37 (23.3)	17 (27.9)	
2010–2014	36 (22.6)	23 (37.7)	
2015–2019	55 (34.6)	12 (19.7)	
Viscetal metastasis at the first diagnosis of breast cancer			
No	66 (41.5)	34 (55.7)	0.0405
Yes	93 (58.5)	27 (44.3)	
Number of metastatic sites			
1	91 (57.2)	49 (80.3)	0.0037
2	45 (28.3)	9 (14.8)	
3≦	23 (14.5)	3 (4.9)	
Tumor subtypes			
HR + /HER2-	91 (59.5)	22 (37.3)	0.0064
HR + /HER2 +	25 (16.3)	8 (13.6)	
HR-/HER2 +	23 (15.0)	19 (32.2)	
TN	14 (9.2)	10 (17.0)	
Tumor subtypes (including unknown)			
HR + /HER2-	91 (57.2)	22 (36.1)	0.0149
HR + /HER2 +	25 (15.7)	8 (13.1)	
HR−/HER2 +	23 (14.5)	19 (31.2)	
TN	14 (8.8)	10 (16.4)	
Unknown	6 (3.8)	10 (17.0)	

*SE* standard error, *HR* hormone receptor, *TN* triple-negative

### Survival analyses

**Fig. 1:**
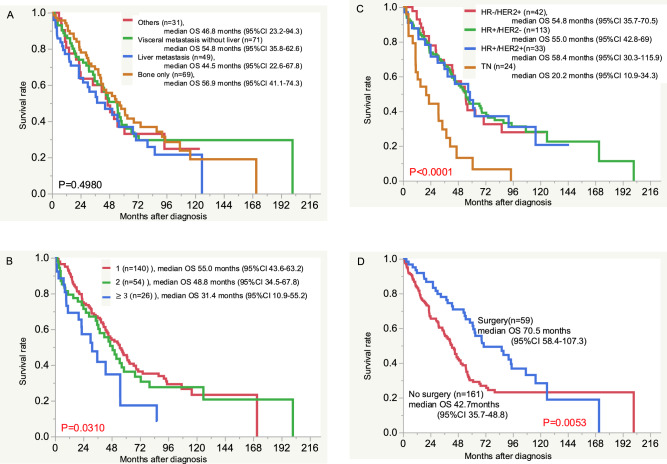
The overall survival of patients according to clinicopathological features. **A** Metastatic sites, **B** Number of the metastatic sites, **C** Tumor subtypes, **D** Surgery for primary site

The median follow-up time was 37.9 (0.5–201.7) months. The median OS was analyzed according to clinicopathological characteristics (Fig. 1). There were no significant differences in the OS according to the metastatic sites (liver, visceral sites other than liver, bone, and other sites) at the first diagnosis (Fig. 1A). The median OS was significantly shorter in patients with ≥ 3 metastatic sites (31.4 months, 95% confidence interval [CI] 10.9–55.2 months) than that of patients with 1 or 2 metastatic sites (55.0 months, 95% CI 43.6–63.2 months; 48.8 months, 95% CI 34.5–67.8 months, P = 0.0310, Fig. 1B). The median OS of the patients with HR + /HER2−, HR + /HER2 + , HR−/HER2 + , and TN tumors was 55.0 (95% CI 42.8–69) months, 58.4 (95% CI 30.3–115.9) months, 54.8 (95% CI 35.7–70.5) months, and 20.2 (95% CI 10.9–34.3) months, respectively. The OS of patients with TN tumors was significantly shorter than that of those with other tumor subtypes (P < 0.0001, Fig. 1C). The OS of the patients who underwent surgery was 70.5 (95% CI 58.4–107.3) months, whereas that of those who did not undergo surgery was 42.7 (95% CI 35.7–48.8) months. The OS was significantly longer in patients who underwent surgery at the primary site than in those who did not (P = 0.0053, Fig. 1D).

**Fig. 2:**
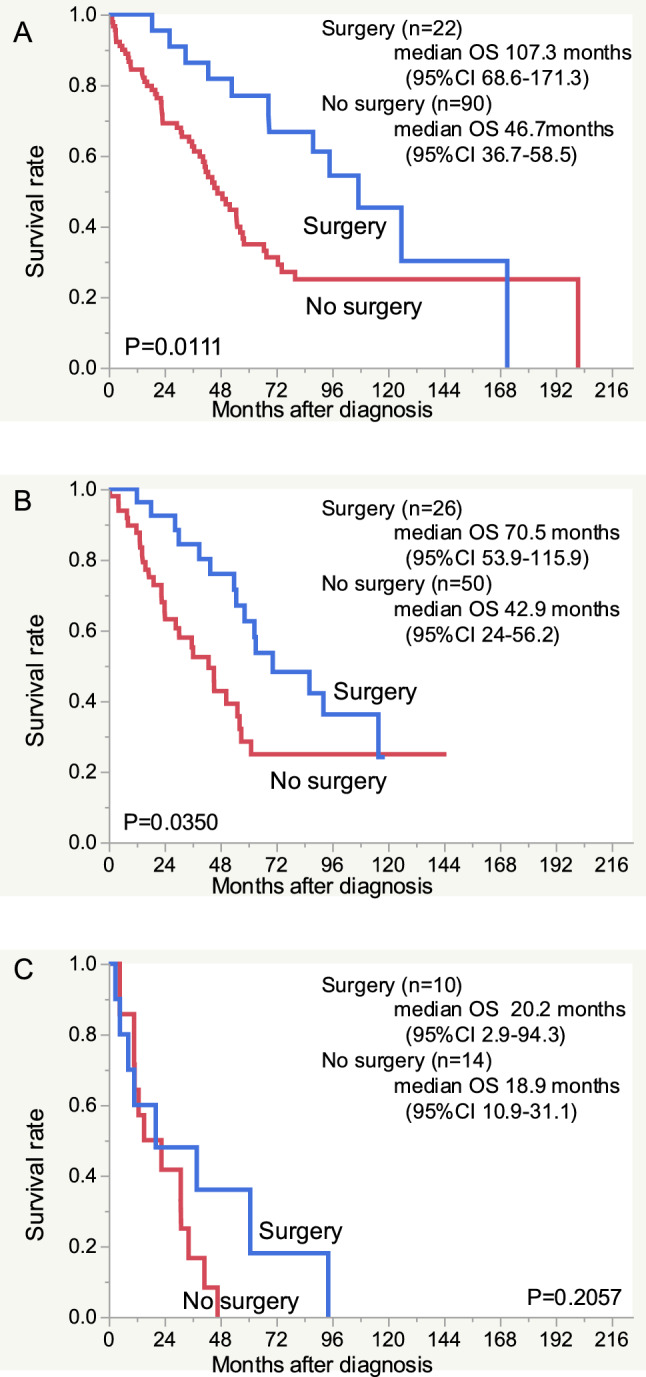
The overall survival of patients with and without surgery for the primary site according to the tumor subtype. **A** HR + /HER2-, **B** HER2 + , **C** TN

The impact of surgery on the OS was analyzed for three subtypes: HR + /HER2−, HER2 + (HR + or HR−), and TN (Fig. 2). In the HR + /HER2− group, the median OS of the patients with or without surgery was 107.3 (95% CI 68.6–171.3) months and 46.7 (95% CI 36.7–58.5) months. In the HER2 + group, median OS of the patients with or without surgery was 70.5 (95% CI 53.9–115.9) months and 42.9 (95% CI 24–56.2) months. Thus, the OS was significantly longer in the patients who underwent surgery for primary site than that in those without surgery in the HR + /HER2− (P = 0.0111, Fig. 2A) and HER2 + subtypes (p = 0.0350, Fig. 2B). Conversely, in the TN group, there was no significant difference in the OS of patients with or without surgery: 20.2 (95% CI 2.9–94.3) months and 18.9 (95% CI 10.9–31.1) months, respectively (Fig. 2C). Pathological results for both biopsy specimens obtained at the start of treatment and surgical specimens were available for 49 patinets. In many cases, the tumor subtypes did not change. However, in some cases, the tumor subtype of the biopsy specimen at the start of treatment differed from the tumor subtype of the surgical specimen. The relationships between these results is shown in Supplemental Table 1.

### Timing of the surgery and OS

**Fig. 3:**
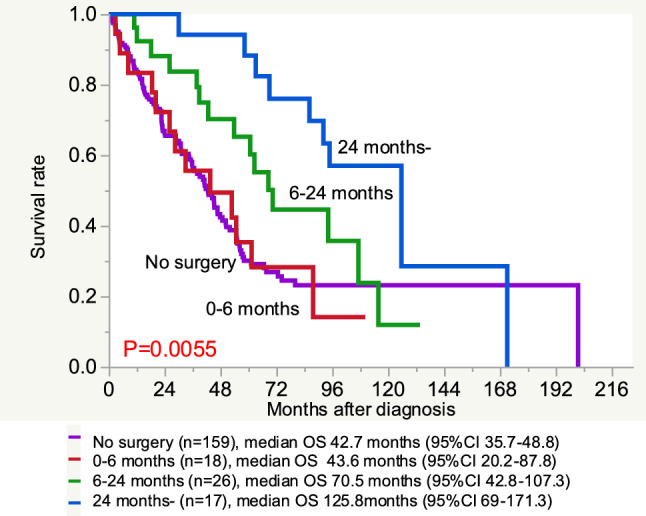
The overall survival of the patients with surgery for primary lesions according to the surgical timing. Patients who underwent surgery at 0 <  < 6 months (0–6 months), 6≦ < 24 months (6–24 months), and ≥ 24 months (24 months-) after the diagnosis

The impact of the surgical timing on the OS was analyzed (Fig. 3). The timing was divided into 3 groups: those who underwent surgery at > 0 to < 6 months, ≥ 6 to < 24 months, and ≥ 24 months after the diagnosis. The median OS was 43.6 (95% CI 20.2–87.8), 70.5 (95% CI 42.8–107.3), and 125.8 (95% CI 69–171.3) months in each group. The median OS of patients who underwent surgery at > 0 to < 6 months was as short as that of patients who did not undergo surgery. The OS was significantly longer in patients who underwent surgery ≥ 24 months after the first diagnosis than earlier (P = 0.0055, Fig. 3). In 16 of the 17 patients (94.1%) who underwent surgery ≥ 24 months, the primary reason for surgery was progression of the primary site only, while all other sites were stable. In contrast, the same reason was found in only 5 (20.8%) and 1 (5.4%) patients who underwent surgery at ≥ 6 to < 24 months and 0 > 0 to < 6 months after the diagnosis, respectively.

### Duration of the first-line ST and the OS

**Fig. 4:**
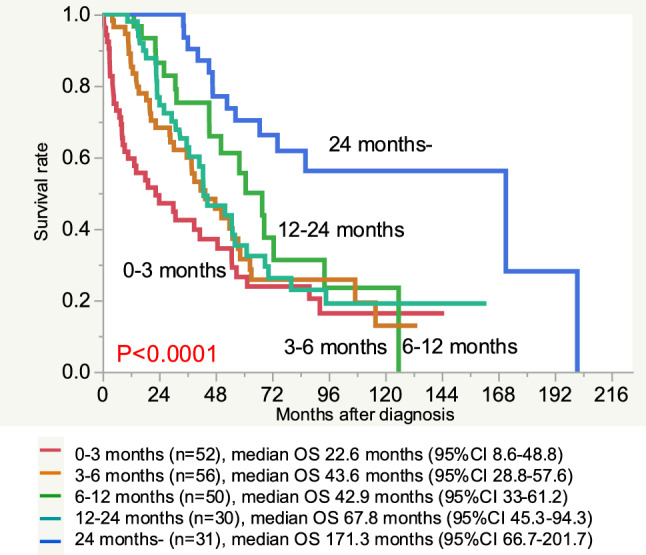
The overall survival and duration of the first-line systemic therapy. Patients who could continue their first-line ST for 0 <  < 3 months (0–3 months), 3≦ < 6 months (3–6 months), 6≦ < 12 months (6–12 months), 12≦ < 24 months (12–24 months), and ≥ 24 months (24 months-)

The relationship between the OS and the duration of the first ST was evaluated (Fig. 4). Among the 220 patients, only 1 patient underwent surgery alone; the other 219 patients received ST. Seven patients underwent surgery immediately after diagnosis, followed by ST. The duration of the first ST after surgery was evaluated for the seven patients who underwent surgery immediately after diagnosis. The median OS was 22.6 (95% CI 8.6–48.8), 43.6 (95% CI 28.8–57.6), 42.9 (95% CI 33–61.2), 67.8 (95% CI 45.3–94.3), and 171.3 (95% CI 66.7–201.7) months in patients who could continue their first-line ST for 0 <  < 3 months, 3≦ < 6 months, 6≦ < 12 months, 12≦ < 24 months, and ≥ 24 months, respectively. The OS in patients in whom the first-line ST could be successfully continued for ≥ 24 months was significantly longer than that in other (P < 0.0001, Fig. 4).

### Univariate and multivariate analyses for the OS

The impact of clinicopathological factors on the OS was evaluated, and univariate and multivariate analyses for the OS were performed. The number of metastatic sites (≥ 3 vs. 1, 2), tumor subtypes (TN vs. others), duration of the first ST ≥ 12 vs. < 12 months, and surgery for the primary site (no vs. yes) were the prognostic factors in both univariate and multivariate analyses. The involvement of visceral metastasis at the time of the diagnosis did not correlate with the OS (Table 3).

**Table 3:** Results of univariate and multivariate analyses for the OS

Factors	Parameters	Univariate analysis			Multivariate analysis		
		HR	95% CI	P-value	HR	95% CI	P-value
Viscetal metastasis at the first diagnosis	Yes vs. No	1.1	0.79–1.56	0.543			
Number of metastatic sites	≥ 3 vs. 1, 2	1.91	1.12–3.08	0.0189	2.07	1.20–3.39	0.0101
Tumor subtypes	TN vs. others	3.08	1.86–4.87	< 0.0001	4.16	2.44–6.79	< 0.0001
Duration of the first systemic therapy	≥ 12 vs. < 12 months	2.32	1.56–3.58	< 0.0001	2.59	1.75–4.19	< 0.0001
Surgery for primary site	No vs. Yes	1.71	1.18–2.54	0.0043	2.02	1.36–3.06	0.0004

*OS* overall survival, *HR* hazard ratio, *CI* confidence interval, *TN* triple-negative

## Discussion

In the present study, we showed that the OS was significantly longer in patients who underwent surgery for primary site with de novo stage IV BC, especially in patients with HR + and/or HER2 + BC, than in those who did not undergo surgery. In addition, the OS of patients with a relatively long duration of first-line ST and those who underwent surgery at ≥ 24 months after the initial diagnosis was the longest. In our cohort, the OS of patients with TNBC was the shortest, and there was no survival benefit from surgery for the primary tumor. These findings emphasize the importance of ST on the survival of patients with de novo stage IV disease and the survival benefit of surgery for the primary site in select patients.

Several randomized studies have been conducted to clarify the importance of surgery for primary tumors in terms of the OS. However, a positive result was observed in only one trial [13]. In that trial, patients were randomized before obtaining knowledge of the HR (ER, PR) or HER2 status; therefore, there were some imbalances between the two arms in terms of the HR status and subtype. These imbalances might have influenced the prognosis regardless of the surgery. However, other studies have failed to show any apparent benefits of surgery for primary sites. In the ECOG-ACRIN 2018 trial, patients who did not progress during four to eight months of ST were randomized to receive loco-regional therapy (LRT) of the primary site plus ST or ST-only continuation. Locoregional progression was less frequent in patients assigned to LRT; however, there was no statistically significant difference in the OS [16]. In two other studies, patients were randomized into two groups: LRT of the primary site plus ST and ST alone. In these studies, the efficacy of ST was not evaluated before LRT [14, 15]. Therefore, it is difficult to discuss the survival benefits of LRT and ST for the OS.

A study using the ESME cohort showed that surgery performed within 12 months of the diagnosis of de novo stage IV BC was associated with a longer OS and PFS [6]. In that study, patients with stage IV BC who were alive and progression-free at 12 months were eligible for an OS evaluation. Surgery performed within 12 months of the diagnosis was associated with fewer than three metastatic sites, treatment with chemotherapy, HER2-targeted therapy, and locoregional radiotherapy. Many patients who died, progressed, or were censored within 12 months after the diagnosis were excluded from the analyses. Therefore, the prognosis was considered better than if these patients had also been included. Another study investigated patterns of surgical care and their association with the OS. Receipt of surgery was independently associated with an improved OS when compared with ST alone, although surgical resection is increasingly frequently being performed after ST [8]. In that study, the authors commented that the greatest benefit was seen among patients with ER + BC who underwent surgery after receiving ST [8]. Our results are similar to those of this study in that ER + BC patients who underwent surgery after receiving ST had a longer OS than those who received surgery without ST or those with other subtypes of BC.

Understanding the beneficial effects of surgery for primary sites on OS in previous studies is quite complicated. The OS duration has varied widely among studies, being shorter in older studies and generally longer in newer ones. This might be largely due to advances in overall treatment strategies [19]. Two impressive reports used data from the NCDB. The median OS of patients was approximately one year without surgery and two years with surgery when diagnosed between 1990 and 1993 [7]. In contrast, the OS of patients diagnosed between 2003 and 2012 was significantly longer than that of patients diagnosed earlier, regardless of whether or not surgery was performed [8]. In the latter NCBD study, among 24,015 patients included in the analyses, 4,552 underwent surgery prior to ST, 5,958 underwent surgery after ST, and 13,505 were treated with ST alone. The median OS was 52.8 months in women who underwent surgery after ST, 49.4 months in women who underwent surgery before ST, and 37.5 months in women with ST alone, respectively. Therefore, selecting the appropriate ST according to the subtype is the most important factor when deciding on a treatment strategy for each patient with MBC. However, the prognosis of MBC differs significantly among the tumor subtypes. For example, the prognosis of HR + or HER2 + BC is significantly better than that of TNBC. ST for MBC has been greatly improved by the emergence of new targeted therapies, including CDK4/6 inhibitors, various anti-HER2 therapies, PARP inhibitors, new antibody–drug conjugates (ADCs), and immune checkpoint inhibitors. Furthermore, the survival improvement in HR + or HER2 + BC was shown to be significantly greater than that in TNBC [19–21]. Median progression-free survival (PFS) differs among subtypes and therapies. In terms of first-line therapy, combination therapy with aromatase inhibitors and CDK4/6 inhibitors provides a PFS longer than two years in HR + /HER2- BC [22, 23]. For HER2 + BC, first-line treatment with pertuzumab, trastuzumab, and taxane achieved a PFS of 18–24 months [24, 25]. Trastuzumab deruxtecan (T-DXd) has shown extremely high efficacy in HER2 + BC even after prior chemotherapy and anti-HER2 therapies [26, 27]. For TNBC, combination therapy with chemotherapy and immune checkpoint inhibitors prolongs the median PFS by seven to nine months [28, 29]. Sufficient time is necessary to evaluate the efficacy of first-line ST for MBC for each patient according to the subtype and the selected treatment. Previous randomized trials designed to clarify the significance of surgery appear to have been inadequate in terms of assessing the efficacy of the first-line ST. It cannot be ruled out that the short duration of ST and its interruption for surgery may have affected the treatment sensitivity and efficacy, which in turn may have affected the OS.

As our data show, primary resection provides a survival benefit for a subset of patients with stage IV BC. However, not all patients benefit from primary tumor resection. The important thing is to first provide the appropriate ST for each patient, evaluate its efficacy, and then fully consider whether primary tumor resection should be performed and, if so, when.

Several limitations associated with the present study warrant mention. All of the data were retrospective, and the number of patients included was small. However, the strength of the present study is that the data were obtained from a single institution with high-quality follow-up and updated clinical data. These results provide an opportunity to fully consider and discuss the benefits of surgery in patients with de novo stage IV BC.

In conclusion, OS was significantly longer in patients who underwent surgery at the primary site than in those who did not. However, it is unlikely that the surgery itself improved the prognosis of all patients. The benefit of surgery was observed in the HR + /HER2- and HER2 + subtypes but not in the TN group. The OS was significantly longer in patients who underwent surgery ≥ 24 months from the first diagnosis than in others, which was correlated with the longer efficacy of the first-line ST. Therefore, the tumor subtypes, efficacy of ST, and timing of surgery could influence the benefits of surgery.

## Supplementary Information

Below is the link to the electronic supplementary material.

Supplementary file1 (XLSX 10 KB)
